# Anxiety and self-efficacy in Chinese international students’ L3 French learning with L2 English and L3 French

**DOI:** 10.3389/fpsyg.2022.998536

**Published:** 2022-12-16

**Authors:** Qiuhan Lin

**Affiliations:** Department of Linguistics and Translation, City University of Hong Kong, Kowloon, Hong Kong SAR, China

**Keywords:** anxiety, self-efficacy, Chinese international students, foreign language acquistion, statistical analysis

## Abstract

The present study explored the relationship between international students’ Third Language Anxiety (TLA) and self-efficacy. The research data were collected through questionnaires involving 243 Chinese International students’ L3 French Learning with L2 English and L3 French at one university in the U.K. Three of them were interviewed about their experience of anxiety and self-efficacy. Major findings include four underlying factors correlated with TLA and two underlying factors correlated with self-efficacy. Also, levels of these students’ TLA were negatively correlated with the level of their self-efficacy, as shown in the correlational analysis. Then, two linear regression models were built to contribute to the prediction of their self-efficacy levels. Lastly, participants reported that grammatical and pronunciation similarities between English (L2) and French (L3) positively decreased their anxiety levels. All of these interviewees encountered communication apprehension. These findings can provide educational implications for L3 teaching and learning, inspiring teachers to consider international students’ TLA and self-efficacy and thus propose some coping strategies.

## Introduction

Foreign Language Anxiety (FLA) is a common phenomenon among foreign language learners since the early 1970s (Santos et al., 2017). It refers to “a distinct complex of self-perceptions, beliefs, feelings, and behaviors related to classroom language learning arising from the uniqueness of the language learning process” (Horwitz et al., 1986, p. 128). It varies among different learners and is affected by multiple factors including linguistics abilities and psychological factors (MacIntyre, 2017).

Previous FLA studies have found that many students encounter FLA (e.g., MacIntyre and Gardner, 1991; Liu, 2006; Liu and Ni, 2015). Some anxious students might engage in self-talk negatively to doubt the ability of his/her own, which hindered them from performing better (MacIntyre and Gardner, 1991). Others might be enmeshed in overstudying: as they were worried about making errors, they attempted to compensate for their errors by studying, but they easily became frustrated once they failed to achieve their expected grades (Horwitz et al., 1986). Hence, these anxious students gain lower self-efficacy (i.e., self-belief that they can master this language) in foreign language learning (Li et al., 2018), and even some of them have a mental block (Tobias, 1979). Therefore, they should be paid attention to. Previous research on anxiety and self-efficacy in language learning has demonstrated a significant correlation, mostly in the context of learning English as an L2. However, scarce studies have measured their relationships in an L3 context. To our knowledge, despite very little literature on the FLA of L3 acquisition, most of it focuses on students’ L3 learning in their motherlands (e.g., Cenoz, 2013; Thompson and Khawaja, 2016; Bensalem and Thompson, 2022). Almost no study has explored international students’ L3 learning in a foreign country with L2 and L3 as the medium. Since international students cannot speak their L1 in their L3 classroom in a foreign country, they might encounter more anxiety and pressure during the L3 learning process, which, in turn, affect their self-efficacy. Thus, their anxiety and self-efficacy in L3 learning should be considered. To fill this gap, this paper is aimed at exploring Chinese international students’ third language anxiety (hereafter: TLA) in a foreign country.

## Literature review

### Foreign language anxiety

Anxiety, associated with people’s nervous system with feelings of tension, worry, nervousness, and apprehension (Spielberger, 1972), has been a common phenomenon among students and a research focus among researchers. Situated in the context of the foreign language learning process, FLA was proposed to explore students’ feelings, behaviors, and self-perceptions when they study a foreign language (Horwitz et al., 1986, p. 127). To be more specific, in the classroom setting, Foreign Language Classroom Anxiety (FLCA) mainly focuses on teaching and learning activities that happen in the foreign language classroom (Horwitz et al., 1986).

Horwitz, the pioneer of the FLA area, proposed the FLCA theory and designed the well-known Foreign Language Classroom Anxiety Scale (FLCAS) with his colleagues (Horwitz et al., 1986). In this paper, FLCA extends over three factors: (1) communication apprehension, (2) test anxiety, and (3) fear of negative evaluation. Communication apprehension refers to one’s shyness to communicate with others due to fear of anxiety, while test anxiety is defined as performance anxiety due to fear of failure, especially in tests. Fear of negative evaluation has a broader scope than that of test anxiety as it occurs in a social, evaluative situation, including apprehension, avoidance, and/or expectations of others’ negative evaluation. After defining these three factors, Horwitz et al. (1986) proposed the FLCAS, which is a 33-item five-point Likert Scale that has been most cited to measure the variable of foreign language anxiety and well-validated by many follow-up studies mainly through factor analysis (e.g., Liu and Huang, 2011; Hasan and Fatimah, 2014; Tsai and Lee, 2018). In addition to the above three-factor solution, follow-up studies using the FLCAS to conduct factor analysis do not have a confirmed classification. They found that there were other classifications of two-factor, four-factor, and five-factor solutions, which were probably due to various experimental and participants’ settings. For example, the two-factor solution involves the factors of low confidence in speaking English and worry about foreign language classroom performance (e.g., Cheng et al., 1999; Liu, 2009). Paredes and Muller-Alouf (2000) proposed four factors, namely, “communication apprehension,” “anxiety about foreign language learning processes and situations,” “comfortableness in using English inside and outside the classroom,” and “negative attitudes towards learning English.”

To measure FLA, collecting questionnaires with statistical analysis based on the FLCAS is one of the most common methods (e.g., Tóth, 2008; Mak, 2011; Bensalem and Thompson, 2022). Apart from this, other instruments for FLA research include interviews, reflective journals, and observations (e.g., Liu and Jackson, 2011; Park and French, 2013; Öztürk and Gürbüz, 2013). These experimental studies in the FLA area have generally found that students’ FLA and their Foreign Language (FL) performance are negatively correlated (e.g., Aida, 1994; Coulombe, 2001; Horwitz, 2001). For example, in 11 French classes, Coulombe (2001) found a weak but significant correlation between students’ FLA and their French grades. In Japanese classes, Aida (1994) and Kitano (2001) also found an inverse correlation between FLA and students’ Japanese performance. In addition to language performance, other variables in the FL learning process, such as age, gender, self-efficacy, motivation, languages, and learning strategies, have also been proven to be correlated with FLA (e.g., Ewald, 2007; Jiang and Dewaele, 2019; Lou and Noels, 2020). These studies all reveal that FLA serves as an independent variable to interact with a multitude of other variables.

### Self-efficacy

Similar to FLA which has been researched since the early 1970s, the concept of self-efficacy was proposed by Bandura (1977). Self-efficacy is defined as “people’s judgment of their capabilities to organize and execute courses of action required to attain designated types of performance” (Bandura, 1986, p. 391). Also, the judgment is situational and task-specific, which means people’s self-efficacy might vary with contexts or tasks (Bandura, 1977).

Regarding the sources of self-efficacy, Bandura (1997) originally proposed four dimensions: mastery experience, vicarious experience, social persuasion, and physiological and psychological state. Mastery experience refers to the personal experience of success (Bandura, 1997), which is the strongest and most authentic evidence for individuals’ belief in their capabilities (Bandura, 1995). Physiological and psychological state is also based on individuals’ inner belief and states, while vicarious experience and social persuasion are based on the belief of others. For example, vicarious experience is watching peers’ success, and social persuasion is about receiving positive evaluations from others (Bandura, 1997). These four dimensions constitute individuals’ self-efficacy.

The main research method of measuring self-efficacy is still questionnaires, sometimes along with interviews, diaries, and observations (e.g., Çubukçu, 2008; Barrows et al., 2013; Torres and Turner, 2016). Although many previous studies have demonstrated that self-efficacy is correlated with students’ academic performance (e.g., Lent et al., 1984; Zimmerman and Martinez-Pons, 1990; Schunk and Swartz, 1991), it is difficult to design a widespread and authority scale as the FLCAS. Researchers in the self-efficacy area have repeatedly highlighted the importance of measuring self-efficacy accurately (Pajares and Miller, 1995; Bandura, 2006; Bong, 2006). Bong (2006) criticized that many self-efficacy scales at that time were inconsistent with Bandura’s (1997) theory, failing to assess self-efficacy.

In light of a few scales of self-efficacy targeted at Chinese students, Da (2006) proposed a self-efficacy scale, a 20-item 5-point Likert Scale, based on Bandura’s (1997) theory. After exploratory factor analysis, self-efficacy was divided into two factors: (1) cognitive engagement; (2) behavior engagement. Cognitive engagement refers to whether students think/believe they can achieve their study aims cognitively, while behavior engagement is defined as students’ subjective judgments of whether they can achieve their learning goals through their actions/behaviors (Da, 2006). Therefore, the present study adopts Da’s (2006) scale to measure self-efficacy. Other researchers also have slightly different interpretations of self-efficacy (e.g., Barrows et al., 2013; Torres and Turner, 2016). For example, Linnenbrink and Pintrich (2003) categorized self-efficacy into behavioral engagement, cognitive engagement, and motivational engagement (referring to interest and utility value). These three factors are all independent, contributing to the self-efficacy field.

### The relationship between anxiety and self-efficacy in foreign language learning

In some empirical studies, the correlation between learners’ FLA and self-efficacy has been measured (e.g., Çubukçu, 2008; Barrows et al., 2013; Torres and Turner, 2016) and demonstrated to be negative (e.g., Bandura, 1997; Matsuda and Gobel, 2004; Barrows et al., 2013). However, this correlation is not fixed, varying with the context. Among Turkish junior students, for example, no correlation between anxiety and self-efficacy was found in Çubukçu’s (2008) study, but Torres and Turner (2016) claimed that Çubukçu’s (2008) study did not consider various difficulty levels of the FL. Additionally, Barrows et al. (2013) revealed that foreign language learners’ anxiety (especially test anxiety) levels and self-efficacy levels could predict their test grades based on linear regression, in which their self-efficacy levels moderated their anxiety levels. Most studies measuring the relationship between anxiety and self-efficacy in foreign language learning focus more on learners’ experience of learning English as an L2 (Bensalem, 2018), whereas few studies have explored the experience of learning an L3 except English as mentioned by Thompson and Lee (2013). Therefore, research on anxiety and self-efficacy in L3 learning should be further investigated.

### FLA and self-efficacy in multilingualism

Multilingualism is defined as “any experience with an L3” by Thompson and Khawaja (2016, p. 1). There have been some relevant studies in terms of anxiety and self-efficacy in the context of multilingualism (e.g., Cenoz, 2013; Thompson and Khawaja, 2016; Bensalem and Thompson, 2022).

Among these multilingualism studies, some studies researched on learning L3 through their L1 (Paredes and Muller-Alouf, 2000; Thompson and Khawaja, 2016). For example, Thompson and Khawaja (2016) explored the foreign language anxiety of using L1 Turkish to learn L3 Spanish. Paredes and Muller-Alouf (2000) investigated the context of using L1 English to learn L3 Spanish, which proposed a Spanish version of the foreign language classroom anxiety scale. There are also other studies exploring combining their L1 and L2 to learn L3 (e.g., Schepens et al., 2016; Mulík and Carrasco-Ortiz, 2021). For example, Mulík and Carrasco-Ortiz (2021) found that phonology in L1 Spanish and L2 English has a positive effect on transferring to learn L3 Slovak. However, there is another group of students learning L3 with L2 and L3, but it was almost no previous study to our knowledge. As many of these students are international students who seldom have a chance to speak L1 in their L3 class in a foreign country, which might cause more anxiety than other students who learn L3 with L1 in their motherland. Therefore, to consider the student group comprehensively, merit particular attention should be paid to this special group of international students, that is, international students learn L3 with L2 and L3 in a foreign country. In terms of the population of this special group, nearly 0.9 million people studied languages except for English in a foreign country in 2014 (ICEF Monitor, 2022), which is a huge population that should be considered. Due to the lack of studies about this large group of international students learning L3 abroad, the exploration of their anxiety in this study is crucial for the realization of their full potential of self-efficacy, which could raise future researchers’ awareness of considering this group’s FLA and self-efficacy.

## The present study

The present study is an experimental study that combines quantitative and qualitative research methods. The quantitative method of questionnaire tends to measure the statistical relationship between Chinese international students’ TLA and self-efficacy, while the qualitative method of interview tends to collect more open-ended data in terms of their authentic experiences and feelings. Below are the research questions (RQ) that this study seeks to answer:

RQ1: How is the anxiety of Chinese international students related to their self-efficacy in learning an L3?
RQ2: How does the anxiety of Chinese international students predict their self-efficacy in learning an L3?
RQ3: How do Chinese international students feel anxiety and self-efficacy when using L2 and L3 to learn an L3?

## Materials and methods

### Participants

The participants of this study were 234 Chinese international postgraduate alumni (120 males and 123 females) who graduated from Newcastle University in the U.K. from 2016 to 2021. These participants were all native speakers of Chinese aged from 24 to 30 (M = 26.1, SD = 0.07). All of them had the experience of using English (L2) and French (L3) to learn French (L3) when attending the University-Wide Language Program (UWLP) at Newcastle University, (2022). In each semester, they needed to take a final exam to evaluate their language abilities. The grade of the final exam (i.e., distinction-band 1, merit-band 2, or pass-band 3) would be shown on their final transcripts for graduation. When they had French classes, they learned French as their only L3 without learning other L3 languages such as Dutch at the same time, and their teachers were native speakers of French. Besides French, which was employed by these teachers to communicate some simple information (e.g., greetings) with students based on their proficiency levels in French, the teachers’ instructed languages included English to explain French vocabulary, grammar, etc. Before the data collection session, these participants were informed of the purpose of this study and the nature of the participants with the contents.

### Measures

#### Background information questionnaire

The participant’s personal information was collected from 3 items of background information, which include age, gender, and language. All of the participants confirmed that English was their L2 language, and French was their L3 language. 13 (5.55%) of them had learned other languages such as Dutch, Japanese, and Korean before, but all of them confirmed that they spent much less time in learning other languages than French. Therefore, French was their L3 and other languages would be their L4, L5, etc.

#### Foreign language classroom anxiety scale

The study adopted the Foreign Language Classroom Anxiety Scale (FLCAS) designed by Horwitz et al. (1986). This scale suits most of the contexts of this study. However, since this study focuses on the group of students using their L2 and L3 to learn an L3, 17 questions were slightly revised from “foreign language” in the FLCAS into “French” to emphasize this context. For example, the first question from the original FLCAS is “I do not worry about making mistakes in my foreign language class.” Considering the French class involved in the specific context of this study, this question was revised into “I do not worry about making mistakes in my French class.” Also, 5 items that do not fit the context of this study were deleted. The remaining 27 items were placed on a 5-point Likert scale (1 = strongly disagree, 2 = disagree, 3 = neither agree nor disagree, 4 = agree, 5 = strongly agree). With an increase in scores, the participants’ anxiety levels rose as well. Once the students completed the questionnaires, their total scores were utilized to divide the participants into three groups: participants with high-level anxiety (range 42–79 score), average-level anxiety (range 80–121 score), or low-level anxiety (range 122–150 score). The range of dividing participants’ anxiety level is based on Marcos-Llinás and Garau’s (2009) paper in 33-item FLCAS, but as the present study deleted 5 items, which were 5 scores, the range in this study is calculated as the range in Marcos-Llinás and Garau’s criteria minus 5. In the present study, there are 91 participants with low-level anxiety, 142 participants with average-level anxiety, and 2 participants with high-level anxiety. Then, to ensure the validity of this slightly revised questionnaire, a Confirmatory Factor Analysis (CFA) was conducted to demonstrate its high internal consistency (Cronbach alpha = 0.889).

#### Self-efficacy scale

This study extracted Da’s (2006) English learning ability efficacy to evaluate the participants’ self-efficacy in learning an L3. Specifically, the questionnaire contains 7 questions, which were also placed on a 5-point Likert scale. Likewise, the scale ranges from 1 to 5 to represent the answers from “strongly disagree” to “strongly agree.” The higher scores they obtained, the higher levels of self-efficacy they had.

#### Semi-structured interview

To understand the participants’ TLA and self-efficacy more comprehensively, 3 participants with different anxiety levels who graduated in 2018 were invited to attend a one-to-one semi-structured interview, including Participant A (PA, high-level anxiety, female), Participant B (PB, low-level anxiety, female), and Participant C (PC, average-level anxiety, male). These three participants were all English-related majors. They self-assessed their anxiety levels as being high, average, and low before the interview. PA and PB majored in Teaching English as a Second or Foreign Language (TESOL), while PC majored in Interpretation. In terms of the language level of courses, the UWLP provided elective courses for students to select the corresponding level by themselves. All of these three participants selected the intermediate-level French course, in which PA achieved the lowest grade (band 3) among the whole class in the final exam, while PC obtained the highest grade (band 1), and PB obtained a medium grade (band 2). Interview questions included their learning experiences, types of anxiety, self-efficacy levels, causes of self-efficacy, and coping strategies. In terms of the language of the interview, all of the interviewees chose to speak Chinese for their convenience. Thus, all of their interview transcripts were translated from Chinese into English and then double-checked by the participants to ensure the translation quality of the transcript.

#### Procedure

This study was conducted in June 2022, including 282 participants who completed the 27-item questionnaire in 20 mins. The questionnaire was manually translated into Chinese and was delivered online through a Chinese questionnaire platform named Wenjuanxing. After each questionnaire was manually checked, 39 questionnaires were classified as invalid ones because of the inconsistency of the participants’ answers.

After collecting the questionnaire data, three participants volunteered to attend the interview. All the interview data was collected online individually through a phone call. Each interview was audio-recorded and lasted for about 15 min.

## Data analysis

Results of the questionnaire were computed using SPSS, which tested the reliability and validity of the statistical analysis. In the process of statistical analysis, the present study conducted: (1) Factor analysis to decompose the scale of “TLA” into four factors and the scale of “self-efficacy” into two factors; (2) Descriptive analysis to describe the general profile of TLA and self-efficacy with their Means and Standard Deviations individually; (3) Correlational analysis to explore the main correlation between TLA and self-efficacy, along with the correlations between TLA’s four independent underlying factors and self-efficacy’s two underlying factors; (4) Linear regression to demonstrate TLA’s applicability to the prediction of the participants’ self-efficacy. In order to analyze the interview data, a thematic analysis was conducted to sort out the main themes according to the information that the interviewees provided. As Braun and Clarke (2006) indicated, thematic analysis is a way of identifying, analyzing, and reporting themes based on qualitative data by using coding. Aiming to categorize similar patterns into the same themes and divide different categories, it includes the following steps: transcribe interview data into transcripts – read transcripts and familiarize with transcript data – generate initial codes – search for themes – review themes (Marks and Yardley, 2012). Hence, it is effective for sorting chaotic data and discovering useful themes for research.

## Results

### The general profile of anxiety and self-efficacy levels among Chinese international students using L2 and L3 to learn an L3

#### Factor analysis of the FLCAS in this questionnaire

Before conducting the statistical analysis, a multivariate normal distribution test was performed. As the sample size is smaller than 5,000, a Shapiro–Wilk test was conducted (*W* = 0.993, *p* = 0.31 > 0.05). Both the skewness and kurtosis values (skewness = 0.156, kurtosis = 0.311) indicated that the data showed a normal distribution. The results of the Kaiser-Meyer-Olkin (KMO) test for the FLCAS and the self-efficacy scale were 0.936 and 0.85, respectively, suggesting that both scales have a high level of validity. A rotated factor analysis (varimax) on the FLCAS generated four factors: Fear of negative evaluation (FLCAS1, 20.55% variance), Communication Apprehension (FLCAS2, 15.28% variance), Fear of Inadequate Performance in the Foreign Language Classroom (FLCAS3, 13.67% variance), and Negative Attitudes towards the English Class (FLCAS4, 12.04% variance), explaining a total of about 61.54% variance. Table 1 below shows the results of the factor analysis of the FLCAS questionnaire in this context.

**Table 1:** Factor analysis of the FLCAS in this study.

Question	FLCAS1	FLCAS2	FLCAS3	FLCAS4	
Q18	0.757^a^				
Q14	0.738				
Q16	0.727				
Q15	0.615				
Q12	0.564				
Q20	0.554				
Q13	0.515				
Q6	0.445				
Q1		0.808			
Q2		0.669			
Q3		0.566			
Q8		0.546			
Q9			0.722		
Q19			0.703		
Q7			0.654		
Q11			0.487		
Q5				0.793	
Q10				0.693	
Q17				0.597	
Q4				0.442	
% of variable	20.55%	15.28%	13.67%		12.04%
% of total variance	61.54%				

As illustrated in Table 1, the first factor FLCAS1 includes Questions 18, 14, 16, 15, 12, 20, 13, and 6 in the questionnaire. It was named based on the situational context, and the factor name “Fear of negative evaluation” was cited by Horwitz et al. (1986) and Mak (2011). The name of the second factor “Communication Apprehension” was cited from Horwitz et al. (1986) and Park (2014), which includes Questions 1, 2, 3, and 8. The third factor “Fear of Inadequate Performance in the Foreign Language Classroom” (Questions 9, 19, 7, 11) and the fourth factor “Negative Attitudes towards the English Class” (Questions 5, 10, 17, and 4) were cited from Tóth (2008) and Mak (2011), respectively. After conducting the factor analysis of the FLCAS, the factor analysis of the other variable of the self-efficacy scale should also be conducted. In terms of the self-efficacy scale, the results of the rotated component matrix are shown in Table 2 below:

**Table 2:** Factor analysis of the self-efficacy scale in this study.

Question	Cognitive engagement	Behavior engagement		
Q24	0.897		
Q22	0.894		
Q23	0.889		
Q25	0.732		
Q27	0.730		
Q26	0.793		
Q21			0.784
% of variable	49.76%	18.4%	
%of total variance 68.17%			

According to Table 2, the first factor of the self-efficacy scale named “cognitive engagement” includes Questions 24, 22, 23, 25, and 27. The second self-efficacy factor named “behavior engagement” includes Questions 26 and 21. These two variables explain a total of about 68.17% variance. Their names were cited from the self-efficacy framework proposed by Linnenbrink and Pintrich (2003).

### Descriptive analysis of the FLCAS and the self-efficacy scale in this questionnaire

In this questionnaire, items 1–20 were aimed at measuring the variable “anxiety” (M = 2.83, SD = 0.78), while items 21–27 were aimed at measuring the variable “self-efficacy” (M = 2.87, SD = 0.74). As shown, both of the participants’ anxiety and self-efficacy levels ranged from a medium one to a high one over the average point of 2.5 on the 5-point Likert scale, in which the participants’ anxiety level (2.83) was slightly lower than their self-efficacy level (2.87). In terms of Standard Deviation (SD), the TLA level had a higher SD than the self-efficacy level, indicating that the participants’ TLA is less clustered around the mean.

### The correlational relationship between the participants’ L3 classroom anxiety and self-efficacy

Table 3 indicates that the participants’ L3 learning anxiety level was negatively correlated with their self-efficacy level (*p* < 0.01). The correlation coefficient was −0.658, suggesting that these two variables were strongly correlated. Also, all of the four factors of the anxiety scale were negatively correlated with the self-efficacy factor with a significant difference, suggesting that these four factors were all correlated with the participants’ self-efficacy and its underlying factor named “cognitive engagement” respectively. As mentioned above, the four underlying factors in anxiety (FLCAS1, FLCAS2, FLCAS3, and FLCAS4) represent “Fear of negative evaluation,” “Communication Apprehension,” “Fear of Inadequate Performance in the Foreign Language Classroom,” and “Negative Attitudes towards the English Class,” respectively.

**Table 3:** The correlational relationship between participants’ anxiety and self-efficacy.

Item	Anxiety	FLCAS1	FLCAS 2	FLCAS 3	FLCAS 4
Self-efficacy	−0.658**	−0.569**	−0.583**	−0.567**	−0.558**
Cognitive engagement	−0.721**	−0.634**	−0.615**	−0.619**	−0.616**
Behavior engagement	−0.016	0.009	−0.066	−0.019	−0.003

** *p* < 0.01.

When selecting the representative values in Table 3, the strongest correlation was between “the participants’ Anxiety and cognitive engagement (Correlational Coefficient = −0.721), while the weakest correlation was between “FLCAS4-Negative Attitudes toward the English class” and “Self-efficacy” (Correlational Coefficient = −0.558). Among these significant correlational relationships, 60% (6 out of 10) of them were strongly correlated (Anxiety with Self-efficacy, Anxiety with cognitive engagement, FLCAS1/2/3/4 with cognitive engagement) as their absolute values of Correlational Coefficient were all larger than 0.6. The other 40% of relationships were moderately correlated due to their absolute values of Correlational Coefficient being larger than 0.5, but smaller than 0.6. All of these correlational relationships in Table 3 were negatively correlated. However, anxiety and its underlying four factors were not correlated with “behavior engagement” (*p* > 0.05), suggesting that the correlations of the factors in anxiety mainly affected the participants’ cognitive engagement instead of behavior engagement among these participants. Due to the limited sample size, this should be further explored in future studies.

### Predictive effects of L3 classroom anxiety on self-efficacy

After completing the correlational analysis, a stepwise regression analysis was conducted to demonstrate whether the predictive effects of the participants’ TLA and its underlying factors can be utilized to build a predicted self-efficacy model. The average of FLCAS1, FLCAS2, FLCAS3, and FLCAS4 was utilized as the predictor variable, while the average of the self-efficacy scale was used as the dependent variable. Table 4 illustrates two models of stepwise linear regression.

**Table 4:** A stepwise regression analysis between anxiety and Self-Efficacy.

	Dependent variable: self-efficacy						
	Item	Beta^a^	*t*-value	*p*-value	Adjusted *R*^2^	VIF	*F*
Model 1	(Constant)		34.246	0.000	0.43	1		183.83**
	Anxiety	−0.658	−13.558	0.000**			
Model 2	(Constant)		34.503	0.000	0.45			1	98.946**
	Anxiety	−1.033	−7.490	0.000**			
	FLCAS1	0.4	2.9	0.004**			

a Beta here refers to the regression coefficients, which indicates the line scope between the Independent variable and the Dependent variable.

** *p* < 0.01.

Model 1 showed that the participants’ anxiety level significantly predicted their self-efficacy as it passed the F test (*F* = 183.83, *p* < 0.01). In this table, the Independent Variable is Anxiety, while the Dependent Variable is Self-efficacy. Therefore, a predicted model of self-efficacy was trained (*p* < 0.001, *α* = 0.05, Adjusted *R*^2^ = 0.43), accounting for around 43% of the total variance of the self-efficacy scale. The Standardized Beta was −0.658 < 0, indicating that with an increase in the participants’ anxiety level, their self-efficacy level decreases. Also, its absolute value of 0.658 suggests that the participants’ anxiety level can predict their self-efficacy level to a large extent. As the VIF was 1, no significant collinearity among variables was detected. Thus, among these participants, their anxiety levels can be used to predict their self-efficacy levels.

Model 2 only includes Anxiety and FLCAS1 (*p* < 0.01). The other constructs within anxiety (i.e., FLCAS 2,3,4) were excluded as their *p* values were larger than 0.05. This modal also passed the F test (*F* = 98.946, *p* < 0.01). This model with “Anxiety” and “FLCAS1-Fear of negative evaluation” as predictor variables (Adjusted *R*^2^ = 0.45) accounted for a higher percentage of the total variance of self-efficacy than Model 1. According to the Standardized Beta, it was interesting to find that the Beta of anxiety was still smaller than 0 (Beta = −1.033), which showed that anxiety is negatively correlated with self-efficacy as in Model 1. However, the Beta of FLCAS1 was larger than 0 (Beta = 0.4), which suggests that students’ fear of negative evaluation is positively correlated with their self-efficacy. Both of these two variables contribute to Model 2, which provides a better model to explain the variable of self-efficacy than Model 1 with a larger Adjusted *R*^2^.

### Interview analysis

After analyzing quantitative data, qualitative data was also collected to illustrate the participants’ experience of anxiety and self-efficacy. In this interview, three interviewees self-evaluated their anxiety and self-efficacy levels. This study utilized thematic analysis to sort out four major themes, which include: (1) differences in teaching different languages; (2) interviewees’ anxiety causes and solutions; (3) interviewees’ self-rated efficacy and underlying factors; (4) interviewees’ suggestions for future learners.

#### Differences between teaching L1 and teaching L2 and L3

After thematic analysis, all three participants reported that the similarity between their instructed languages and target languages in grammatical rules and pronunciation rules influenced their TLA. For example, PC (average-level anxiety, male, band 1) showed a positive attitude towards the similarities between English and French, believing that he would understand French better with English than with Chinese, whereas PA (high-level anxiety, female, band 3) held a negative opinion, indicating that it would be easier for her to confuse English with French.

#### Anxiety types and corresponding solutions

In terms of anxiety types, all three participants mentioned communication apprehension when talking to native speakers. They feared being ridiculed by natives because of their accents. Liu (2006) also found this communication anxiety in her interview when Chinese students spoke English to others at different English proficiency levels. Similarly, in this study, PC (average-level anxiety, male, band 1) even worried that his French could not be as fluent as his English. Especially when he was traveling in France, he could not use French for daily communication. Only PA (high-level anxiety, female, band 3) had test anxiety, worrying that she would fail the exam and could not graduate successfully. To overcome anxiety, PC (average-level anxiety, male, band 1) had an active mind that learning French was just for his interest. PA (high-level anxiety, female, band 3) tried to transform pressure into motivation. When considering the possible negative consequences of failing the exam, she forced herself to go to the class, even if it was very painful for her. “Every time when the French class was over, I felt a big relief. But our teacher was very nice, so the French class was not as terrifying as I imagined,” PA reported. She also confirmed the teacher’s quality of “being nice” had helped her relieve a lot. It is in line with the findings of previous research that Teaching English as a Foreign Language (EFL) teachers are advised to create a relaxing and supportive classroom atmosphere (Zou, 2004; Liu and Jackson, 2009).

#### Participants’ self-efficacy of the L3 ability and its corresponding factors

When the three participants were invited to self-evaluate their L3 ability from 1 to 10 (1 is the worst; 10 is the best), PC (average-level anxiety, male, band 1) gave himself a score of 6, PA (high-level anxiety, female, band 3) gave herself only a score of 2, and PB (low-level anxiety, female, band 2) gave herself a lower or middle score of around 4 to 5. Regarding the factors influencing their self-efficacy, all of them considered their French performance in real-life communication. PA considered another factor in test grades.

#### Suggestions for future students

The last interview question is whether they have any suggestions for other future students to use L2 and L3 for L3 learning if they also register for this course. PC (average-level anxiety, male, band 1) focused more on the attitude of learning French: “I would say if you want to improve your French, you have to learn it very consistently because I do not think there is any shortcut in language learning. My suggestion is that do not lose heart when you feel frustrated. If you do not give up, if you keep learning and learning, your French will be improved.” PA (high-level anxiety, female, band1) emphasized that future students need to compare the differences between the L2 and the L3, considering this very carefully. For example, it is easy to confuse English with French.

## Discussion

In the present study, both the participants’ TLA and self-efficacy were at a medium to a high level over a 5-point Likert scale, in which their TLA levels were slightly lower than their self-efficacy levels. Their TLA levels were negatively correlated with their self-efficacy levels in L3 learning. This is in line with the research findings by Haley et al. (2014) and Bensalem (2018). They have found that the FLA levels of non-native speakers are negatively correlated with their self-efficacy levels, but they are learners who learn English as their L2 instead of L3. The reason for this correlation could be that FLA would distract L2 learners’ attention and consume their energy to focus on a task (Gardner et al., 1993). Then FLA would become a cause of some students’ low grades in language learning with low achievement (Horwitz, 2001, 2010; Awan et al., 2010), thereby affecting students’ self-efficacy (Barrows et al., 2013; Dull et al., 2015). For example, in Barrows et al.’s (2013) study, a significant negative correlation was found between students’ FLA and test scores. Meanwhile, a significant positive correlation was found between students’ self-efficacy and test scores. Lastly, in this study, FLA and self-efficacy can be utilized to predict their academic performance (i.e., test scores) by linear regression.

A factor analysis was conducted to examine the underlying factors of TLA and self-efficacy. Results showed that four factors were devised, including “fear of negative evaluation,” “communication apprehension,” “fear of inadequate performance in foreign language classes,” and “negative attitudes towards the English class.” The factors “fear of negative evaluation” and “communication apprehension” are similar to Horwitz et al.’s (1986) theory. In addition, the factor “fear of inadequate performance in foreign language classes” was cited by Tóth (2008). The other factor “negative attitudes toward the English class,” was mentioned by both Tóth (2008) and Mak (2011). Also, other scholars have put forward other classifications of factors. For example, Bensalem and Thompson (2022) proposed two factors “anxiety” and “self-confidence.” Therefore, the four-factor solutions in the present study are consistent with some of the previous research (e.g., Horwitz et al., 1986; Tóth, 2008; Mak, 2011), but there are various types of division for the underlying factors that affect FLA (e.g., Bensalem and Thompson, 2022). Thus, there is no fixed answer to the division of factors in FLA, which varies from context to context.

To answer RQ1, in terms of correlations, this study was in line with previous studies (e.g., Haley et al., 2014; Li et al., 2018), as participants’ learning anxiety was negatively correlated with their self-efficacy, although these studies targeted at students who learned English as an L2. This can be explained by the broaden-and-build theory (Fredrickson and Joiner, 2018), in which negative emotions, including anxiety, tend to cause negative effects (Dewaele and Li, 2021; Dong et al., 2022). Therefore, students are advised to reduce their anxiety to gain higher self-efficacy. To answer RQ3, the stepwise linear regression built two models to predict self-efficacy, which Model 2 is better. A similar study is by Li et al.’s (2018), which built a regression model for FLA to predict participants’ self-efficacy with the R square of 0.33. This R square was similar to the present study’s R square of 0.43 in Model 1, which could suggest that these participants’ anxiety can predict around 30 to 45% of their self-efficacy variance. This can be interpreted as: in Bandura’s (1997) theory, psychological states are one of the four sources that contribute to self-efficacy. Anxiety, one of the psychological states, plays an important role in affecting students’ self-efficacy, which accounts for almost 1/3 to 1/2 of its variance. The other three sources might affect self-efficacy, as mentioned in the literature review session of Bandura’s (1997) theory. However, the impact of anxiety that affects self-efficacy cannot be ignored.

Then, qualitative analysis of interview data answered RQ3. As non-native speakers, they were anxious when communicating with native speakers, with the fear of being ridiculed by their native teachers and/or native classmates. This could be explained by language shock, which was defined by Stengel (1939) that it referred to an individual’s lack of language competence to express his/her idea correctly in a nonnative language. After that, Miranda and Umhoefer (1998) explained that language shock would create stress for L2 learners in their verbalization process and cause them to undermine their self-efficacy due to their fear of making mistakes, thereby impeding their cognitive process. Other scholars, such as Haley et al. (2014), might show another explanation for their identity as non-native speakers. Based on their experiments’ findings, non-native English speakers have significantly higher levels of FLA than native English speakers. Therefore, the nonnative identity of students in the present study might also affect their anxiety level.

Besides, “communication apprehension” was common among these three participants. Furthermore, two of them preferred the current learning mode of combining English and French. Both of them mentioned the positive role of this mode in helping them better understand French due to the similarity between English and French in their grammatical structures. This echoes the findings of some previous studies that, theoretically, the positive language interaction between foreign languages studied (PPLI) can help students be aware of the language interactions among their multiple languages and interpret their dynamic nature (Thompson, 2013). Similar results have been found from Thompson and Khawaja’s (2016) two interviewees, indicating that their experience of learning English as L2 can help them understand an L3.

## Conclusion

To conclude, the present study has explored the relationship between FLA and self-efficacy levels in international students’ use of L2 English and L3 French to learn L3 French abroad. The findings of this study showed that in L3 learning, many participants experienced a medium to a high level of anxiety. Their anxiety levels were negatively correlated with their self-efficacy levels. This tendency is similar to what L2 learning research has generally found. Besides, two regression models to predict the level of self-efficacy were built, in which the combination of anxiety and FLCAS1 can help to predict the level of self-efficacy better. Also, some solutions to lowering the level of FLA have been suggested in this paper, which provides educational implications for teachers to pay more attention to international students’ anxiety and self-efficacy.

Nevertheless, this paper still has some limitations. The first limitation is the selection of the participants. Since it is difficult to find a group of international students who use both L2 and L3, alumni were invited to participate in this study. Some of them even graduated 4 years ago, which might affect their choices in the questionnaire. Secondly, the participants were sampled from only one university, which might affect the homogeneity in the region. Therefore, this paper calls for more attention to international students learning L3 with FL, which should be further studied.

## Data availability statement

The original contributions presented in the study are included in the article/Supplementary material, further inquiries can be directed to the corresponding author.

## Ethics statement

Written informed consent was obtained from the individual(s) for the publication of any potentially identifiable images or data included in this article.

## Author contributions

The author confirms being the sole contributor of this work and has approved it for publication.

## Conflict of interest

The author declares that the research was conducted in the absence of any commercial or financial relationships that could be construed as a potential conflict of interest.

## Publisher’s note

All claims expressed in this article are solely those of the authors and do not necessarily represent those of their affiliated organizations, or those of the publisher, the editors and the reviewers. Any product that may be evaluated in this article, or claim that may be made by its manufacturer, is not guaranteed or endorsed by the publisher.

## Supplementary material

The Supplementary material for this article can be found online at: https://www.frontiersin.org/articles/10.3389/fpsyg.2022.998536/full#supplementary-material
