# Supplementary material for: Anxiety and self-efficacy in Chinese international students’ L3 French learning with L2 English and L3 French
Source: Front Psychol. 2022 Dec 16;13:998536. doi: 10.3389/fpsyg.2022.998536 (PMC9800968; doi:10.3389/fpsyg.2022.998536)
Supplement: Supplementary file 1 [file Data_Sheet_1.DOCX]

**Interview questions**

1. Can you briefly **describe** your experience of using English to learn a third language like French？
2. Is there any **difference** between **using the first language to learn French and using a second language to learn French**? Which way do you **prefer?**
3. Did you encounter any **anxiety** when using English to learn French? How did you **navigate these anxiety emotions**?
4. Do you believe that you can learn French very well? How will you quantify your French ability from 1 to 10 (1 is the worst, 10 is the best)? What factors will affect your self-efficacy?
5. Do you have any **suggestions** for those students who use a second language to learn a third language?

Interview question

1. 请简要描述一下您用英法双语学习法语的经历故事；

**我是在2017到2018年就读纽卡斯尔大学的研究生，在读研的过程中参与了第二外语的课程，我选择了法语。我们的老师是一个很nice的法国人，因为我们班有来自不同国家的学生，所以她会尽力照顾到每个人。基本上课堂上她都用她带有法国口音的英语上课，介绍新单词的时候会先用英语讲一遍这个单词是什么意思，然后再用法语读一遍这个单词怎么读，如果我们读不懂，她还会再用英语再解释一遍。**

1. 您觉得用母语学法语，和用英法双语学法语是否存在区别呢？您更喜欢哪种方式呢？

**我觉得用母语学法语的话，很多你不懂的点可以无障碍跟老师沟通，但是如果用英法双语学法语的话，脑子里需要先过一遍中文，然后再翻译成英语，总有一些困难就是表达不出来然后就干脆沉默不想表达了，而且有些法语单词太像英文了，又有点不一样，很容易搞混，我还是更喜欢用母语学法语。**

1. 你在用英法双语学法语的过程中，有感受到焦虑的情绪吗？一般是什么类型的焦虑呢？您是如何处理这种焦虑情绪的呢？

**有啊，我特别焦虑，因为我是我们班的学渣。我们班还有一个中国的同学是学霸，他是参过军的他特别自律，每天六点钟就起来读法语了。我的法语跟同学比起来就更自卑了，特别怕同学嘲笑我。然后我是很怕我法语会挂科，到时候毕业证上要是显示出来就不好了，所以我特别担心我的考试。而且我那个法语老师也特别照顾我，考试的时候老师老是鼓励我，不过我还是跟老师沟通的时候有点拘谨，一是怕我的法语太烂了老师是native speaker会笑我，但是有的真的表达不出来啊，二是真的有的时候表达觉得老师也不一定能get到我的点，于是就算了。**

**我一般是考试上的焦虑居多吧，但就是和老师沟通上的焦虑也有，反正就是上这个法语课好焦虑，每周周五都不想去上课，只能逼着自己硬着头皮去上，不上就有可能会挂科啊，所以只能把压力变成动力了。但是每次上完法语课，总有一种如释重负的感觉，而且老师很nice啊，也没想象中那么可怕吧。**

1. 您相信自己可以成为一个很擅长学习法语的人吗？如果用1-10分评估你的对掌握法语的自信程度（在刚上完法语课之后），您会给自己打几分呢？1最低，10最高。您刚在评估，考虑到什么因素？

**不是吧，我觉得我并不是擅长法语，甚至我学法语的时候容易和英语搞混。我的法语水平也就2吧，主要考虑到我能不能自信顺畅的跟别人沟通，能不能考个好成绩咯。**

1. 如果将来也有学生用第二和第三语言结合（英语）学习第三语言（法语），您会向他们提供什么建议呢？

**我觉得大家可能先多接触先多试几节课再报名吧，虽然用英法双语学法语看起来很高大上，但是很容易搞混啊。特别是英语和法语长那么像，需要分清他们之间的区别。如果可以还有一个中国教师可以补习的话，说不定母语学法语再加上英语学法语会更好。**
