# Supplementary material for: Anxiety and self-efficacy in Chinese international students’ L3 French learning with L2 English and L3 French
Source: Front Psychol. 2022 Dec 16;13:998536. doi: 10.3389/fpsyg.2022.998536 (PMC9800968; doi:10.3389/fpsyg.2022.998536)
Supplement: Supplementary file 2 [file Data_Sheet_2.DOCX]

1. Please briefly describe your experience

I learnt French at Newcastle University in 2017-2018, and I took a second foreign language course during my postgraduate study, and I choseOur teacher is a veryniceFrench, because we have students from different countries in our class, so she will try her best to take care of everyone. Basically, she uses her English with a French accent in the class. When introducing new words, she will first say what the word means in English, and then read the word in French. If we can't understand it, She will explain it again in English.

2. Do you think there is any difference between learning French in your native language and learning French in English and French? Which way do you prefer?

I think that if you learn French in your native language, you can communicate with the teacher without any problems, but if you learn French in English and French, you need to read Chinese in your mind first, and then translate it into English. There are always some difficulties in expressing If I don’t come out, I just keep silent and don’t want to express myself. Moreover, some French words are too similar to English, and they are a little different.

3. Did you feel anxious when you were learning French in both English and French?

What type of anxiety is it? How do you deal with this anxiety?

Yes, I am very anxious because I am not a top student in our class. There is also a Chinese classmate in our class who is a scholar. He has been in the army and he is very self-disciplined. He gets up at six o'clock every day to read French. Compared with my classmates, my French is even more inferior, and I am especially afraid that my classmates will laugh at me. Then I was very afraid that I would fail the French class, and it would be bad if it was displayed on the diploma, so I was particularly worried about my exam. And my French teacher also took special care of me. During the exam, the teacher always encouraged me, but I was still a little cautious when communicating with the teacher. First, I was afraid that my French was too bad. The teacher would laugh at me as anative speaker, but some I really can't express it, and secondly, I sometimes express that I feel that the teacher may not be able togetmy point, so forget it.

I am usually most anxious about exams, but I also have anxiety about communicating with teachers. Anyway, I am so anxious about taking this French class. There is a possibility that he will fail the course, so he can only turn the pressure into motivation. But every time I finish a French class, I always feel a sense of relief, and the teacher is verynice, not as scary as I imagined.

4. Do you believe you can become a very good French learner? If there were 1-10 to rate your confidence in mastering French, how would you rate yourself? 1 is the lowest, 10 is the highest. What factors did you consider when you were rating?

No, I don't think I'm good at French, and even when I learn French it's easy to get confused with English. My French level is only2, mainly considering whether I can communicate with others confidently and smoothly, and whether I can get good grades in the test.

5. If there were any other student who joins this class in the future to use L2 and L3 for studying L3, what suggestions will you give to them?

I think you might get in touch and try a few more classes before signing up. Although learning French in both English and French seems very high-level, it is easy to get confused. In particular, English and French are so similar in length, and it is necessary to distinguish the difference between them. If there is a Chinese teacher who can tutor, maybe it will be better to learn French as a mother tongue and learn French in English.
