# Supplementary material for: Anxiety and self-efficacy in Chinese international students’ L3 French learning with L2 English and L3 French
Source: Front Psychol. 2022 Dec 16;13:998536. doi: 10.3389/fpsyg.2022.998536 (PMC9800968; doi:10.3389/fpsyg.2022.998536)
Supplement: Supplementary file 3 [file Data_Sheet_3.DOCX]

2022年7月1日晚上8:16

提问： 好的。现在开始我们的采访。感谢您参加我们的面试，我们想做一个关于探索学生焦虑水平的研究，首先可以我问一个问题，你介意简单描述一下你用英法双语学习像法语这样的第三种语言的经历吗？

回答：好的。我在大学里主修英语语言和文学，那是在2005年到2009年之间。我开始用英语和法语双语结合学习法语，那是在从2017到2018年期间，我在纽卡斯尔大学学习翻译和口译专业研究生的时候。所以算起来我已经用英语学习法语四年了。

提问：所以在你的本科期间，你是通过普通话还是英语，或者其他语言学习法语呢？

回答：老师用的普通话教学法语。

提问： 那你觉得用母语学法语，和用英法双语学法语， 两者之间有什么区别吗？你更喜欢哪种方式呢？

回答： 是的。我认为有不少差异。例如，与中文相比，英语在语法上更接近法语。对于例如，两种语言都有相似的规则。规则比英语复杂得多。但是，在中文中我们没有动词。所以我认为从英语学习中获得的一些关于类似的知识使我的法语学习更快更有效。其次，一些英语单词在拼写和含义上与法语非常相似。所以这意味着当我开始用英语学习法语时，我已经有了一些法语词汇作为基础。所以与中文相比。我更喜欢用英语来学习法语，因为它让我的学习过程更轻松。

提问： 好的好的，那你在用英法双语学习法语的时候，有没有感觉到焦虑呢？

回答：我会说是的，我认为我最大的焦虑是。我想知道什么时候我的法语可能和我的英语一样好，因为从我的角度来看。法语本身比英语就难学得多。即使我是口译专业的，我可以流利地说和写英语，但是我不可能把我的法语练成这个样子。所以我最大的焦虑是希望我的法语可以练得和我的英语一样好。我的焦虑更多是关于如何用法语和母语者流畅的沟通。你知道要通过考试对我来说并不是一个大问题，实际上我的法语考试考得非常好。但是当我开始用法语和别人说话，我真的很沮丧，因为大多数时候我说不出话来。即使我的同龄人和我几乎处于同一水平，我们只是用非常简单的法语单词相互交流。我仍然感到非常沮丧，我记得我去巴黎的时候，我试图用法语和当地人交谈。但每次我只能说这样的话，我相信。但我无法与当地法语进行适当的对话。所以我经常开始用法语说话，然后大约一分钟左右我会转到英语。

提问：我完全可以理解，所以你有没有尝试过任何方法来驾驭这种焦虑情绪？你之前是如何解决这些问题的?好的对我来说？

回答：你知道，因为我没有学法语专业。我不需要依靠法语谋生，所以我告诉自己我学法语只是为了好玩。如果我能学好法语，那将是一个非常好的事情。如果我做不到，那对我来说是一个兴趣。这样可以让我知道更多地关注学习法语的部分，但是却忘记了焦虑这部分，所以我学习法语只是为了我的兴趣。

提问：你相信你法语能学得很好吗？从一到十如何量化你的能力？

回答：我想如果我能坚持不懈地学习法语，更认真地学习法语，我会学得很好。但是你知道我不是很专注。你知道我只是为了好玩才学的，所以我不是很认真。所以我真的不知道如何识别我的法语水平。我会说我会给自己打六分，我想我认为至少我应该通过，因为我在法语测试中确实做得很好，虽然在真正的谈话中我经常感到沮丧。但无论如何，因为我已经付出了很多努力，我会给自己一个六分。

提问：所以当你刚刚评估时，其实你评估的是你的自我效能。那么你认为哪些因素会影响你的自我效能呢？

回答：嗯，好的。我觉得我还可以，所以我会给自己一个及格的。但在现实生活中的沟通方面我不是很好。所以我把这沟通时的焦虑和考试的焦虑两个因素结合在一起，给自己一个及格。你知道如果我能花更多的时间在学习上，我会给自己一个更好的成绩。

提问：如果我们换另一个中国老师教法语，你觉得会影响你自己，让你变得更有信心说法语吗？

回答：其实我在读本科时，我的法语老师是一位中国女士，而当我在纽卡斯尔学习法语时，这位女士是一位法国本地人。是的，所以我会说。我认为确实有区别。因为当你和你的本地人交谈时法语老师你有时会这样，因为她是本地人，而你不是本地人。你会这样。如果你犯了错误，她可能会嘲笑你，尽管她不会因为她是她母语者就怎么样。她是很好的老师，当我在读学士学位，因为我知道她不是本地人，所以她的发音也可能也有一些问题。所以我想说我觉得更安全一些，而且大部分时间她实际使用和我们说话的时候还是以普通话和法语为主。但是在我在纽卡斯尔的法语课上，老师想方设法给我们更多的法语输入，所以大部分时间她会说法语。所以我会说这差别不大。

提问：是的，是的。我可以明白了。是的。这对我很有帮助，很抱歉。最后一个问题是。所以如果我们将来有更多的学生用英法双语学习法语，你也有什么建议让那些学生喜欢提高他们的经验或学习法语的效率吗？

回答：我认为你必须这样做。你需要为你的学习树立一种学习态度，无论你是想认真学习还是只是为了好玩而学习。我的意思是，如果你真的想把你的法语提高到非常高的水平，你必须非常敬业。不像我，我研究了一段时间我把它放在一边，然后我再研究一次。但是现在法语已经忘得差不多了。所以我想说，如果你想真正提高你的法语。你必须非常一致地学习它，因为我认为语言学习没有任何捷径。如果你想掌握一门语言，你必须坚持下去。我的建议是，即使你感到沮丧，也不要灰心。即使你有那种感觉。你无法将它学习到高水平。如果你不放弃。如果你坚持学习和学习，你会进步的。

提问：好的。谢谢你参加我们的面试。这些答案真的很有帮助，我们的面试就这样结束了。谢谢你。
