# Supplementary material for: Anxiety and self-efficacy in Chinese international students’ L3 French learning with L2 English and L3 French
Source: Front Psychol. 2022 Dec 16;13:998536. doi: 10.3389/fpsyg.2022.998536 (PMC9800968; doi:10.3389/fpsyg.2022.998536)
Supplement: Supplementary file 4 [file Data_Sheet_4.DOCX]

Interview on July 1, 2022 at 8:16pm

Question: OK. Now start our interview. Thank you for participating in our interview, we would like to do a study on exploring students' anxiety levels, may I ask a question first, would you mind briefly describing your experience of learning a third language like French in English and French?

Answer: OK. I majored in English Language and Literature in college, which was between 2005 and 2009. I started bilingually studying French in English and French when I was a postgraduate student in Translation and Interpreting at Newcastle University from 2017 to 2018. So I've been learning French in English for four years now.

Question: So during your undergraduate years, did you learn French through Mandarin or English, or any other language?

Answer: The teacher uses Mandarin to teach French.

Question: Do you think there is any difference between learning French in your native language and learning French in English and French? Which way do you prefer?

Answer: Yes. I think there are quite a few differences. For example, English is grammatically closer to French than Chinese. For example, both languages ​​have similar rules. The rules are much more complicated than in English. However, in Chinese we have no verbs. So I think some similar knowledge gained from learning English made my French learning faster and more effective. Second, some English words are very similar to French in spelling and meaning. So this means that when I start learning French in English, I already have some French vocabulary as a foundation. So compared to Chinese. I prefer to learn French in English because it makes my learning process easier.

Question: Okay, did you feel anxious when you were learning French in both English and French?

Answer: I would say yes, I think my biggest anxiety is. I wonder when my French might be as good as my English because from my point of view. French itself is much more difficult to learn than English. Even though I'm an interpreter major, I can speak and write English fluently, but it's impossible for me to develop my French like this. So my biggest anxiety is hoping my French can be as good as my English. My anxiety is more about how to communicate fluently with native speakers in French. You know it's not a big problem for me to pass the test, I actually did very well on the French test. But when I started talking to people in French, I was really frustrated because most of the time I was speechless. Even though my peers and I are almost at the same level, we just use very simple French words to communicate with each other. I'm still very frustrated, I remember when I went to Paris, I tried to talk to the locals in French. But every time I can only say something like that, I believe. But I can't have a proper conversation with the local French. So I often start speaking in French and then in about a minute or so I switch to English.

Question: I totally get it, so have you tried anything to manage this anxiety? How did you solve these problems before? Okay for me?

Answer: You know, because I didn't major in French. I don't need to rely on French for a living, so I tell myself I'm learning French just for fun. It would be a very good thing if I could learn French well. If I can't, it's an interest for me. This allows me to know to focus more on the learning French part, but forget about the anxiety part, so I learn French just for my interest.

Question: Do you believe you can learn French well? How do you quantify your abilities from one to ten?

Answer: I think if I can study French consistently and more seriously, I will do well. But you know I'm not very focused. You know I just learned it for fun, so I'm not very serious. So I really don't know how to identify my French level. I would say I would give myself a six, I think I think at least I should pass because I did really well in the French test, although I was often frustrated in real conversations. But anyway, because I've put in a lot of effort, I'll give myself a six.

Question: So when you're just evaluating, you're actually evaluating your self-efficacy. So what factors do you think influence your self-efficacy?

Answer: Well, okay. I think I'm okay, so I'll give myself a pass. But I'm not very good at communication in real life. So I combined the anxiety of the communication and the anxiety of the exam together to give myself a pass. You know if I could spend more time studying, I would give myself a better grade.

Question: If we switch to another Chinese teacher to teach French, do you think it will affect yourself and make you more confident to speak French?

Answer: Actually, my French teacher was a Chinese lady when I was an undergraduate, and when I was studying French in Newcastle, the lady was a French native. Yes, so I will say. I think there is indeed a difference. Because when you talk to your native French teacher you sometimes do because she is a native and you are not a native. you will. If you make a mistake, she might laugh at you, although she won't do it just because she's her native speaker. She's a great teacher, and when I was doing my bachelor's degree, because I know she's not local, she might have some problems with her pronunciation too. So I would say that I feel safer, and most of the time she actually uses Mandarin and French when she actually speaks to us. But in my French class in Newcastle, the teacher managed to give us more input in French, so she spoke French most of the time. So I'd say it's not much of a difference.

Question: Yes, yes. I can see. Yes. This helped me a lot, sorry about that. The last question is. So if we have more students learning French bilingually in English and French in the future, do you also have any suggestions for those students who would like to improve their experience or learn French efficiently?

Answer: I think you have to. You need to develop a learning attitude for your studies, whether you want to study seriously or just for fun. I mean, if you really want to improve your French to a very high level, you have to be very dedicated. Unlike me, I researched it for a while I put it aside and then I researched it again. But French has almost been forgotten by now. So I would say if you want to really improve your French. You have to learn it very consistently because I don't think there are any shortcuts to language learning. If you want to master a language, you have to stick with it. My advice is, even if you're feeling down, don't get discouraged. Even if you feel that way. You can't learn it to a high level. If you don't give up. If you keep studying and learning, you will improve.

Question: OK. Thank you for participating in our interview. The answers were really helpful and that's the end of our interview. Thank you.
