# Supplementary material for: Anxiety and self-efficacy in Chinese international students’ L3 French learning with L2 English and L3 French
Source: Front Psychol. 2022 Dec 16;13:998536. doi: 10.3389/fpsyg.2022.998536 (PMC9800968; doi:10.3389/fpsyg.2022.998536)
Supplement: Supplementary file 5 [file Data_Sheet_5.DOCX]

2022年7月1日 晚上8:50

提问：好，那我们的采访开始了，首先非常谢谢你参与我们的采访。那么我们这个采访是全程是匿名的，而且你的信息将会被保密，可以请你简要的描述一下，就是你用英法双语学习法语的一些经历故事吗？

回答：呃，我用英语学习法语是我在上研究生的时候，利用课余直接去参加学校的一个学习项目。嗯，当时我的法语老师是一个来自法国的母语者。呃，因为我们学的那门课程是法语入门，所以他教给我们的是一些比较基础的法语知识。比如说法语的发音，比如说一些简单的表达法。呃。老师上课的时候还是比较注重口语表达的。嗯，这是我学习法语的一些小经历。

提问：ok，那你觉得就是用母语去学法语。比如说你用普通话去学法语，和你用英法双语去学法语的时候，有没有一些什么样的区别呢？

回答：呃，还是有区别的。我觉得用第二语言，特别是英语去学习法语的话其实是会有一些帮助的。当时我在英国我学习法语这门课程的时候，我记得我们的老师在上课的时候都用国际音标来帮助我们去学习。呃，这个方法其实是对于我们来说非常的有效。如果我们使用我们的母语中文去学习法语的话，这里可能会存在一些呃语言的一些差异，对，这个可能还是用英语去学习比较比较好一点，方便一点。对，比较好，对。

提问：ok，那你其实就是更prefer说我是用英法双语来学法语，还是用普通话学法语呢？

回答：啊，我比较喜欢用英法双语学法语。

提问：那你在用英语学法语的时候有没有感受到一些焦虑的情绪呢？

回答：呃，其实是比较少的。因为我在学这门课程之前，其实我自己是有一定的法语基础，然后老师教的又是比较基础的法语比较入门的一些知识。所以焦虑是比较少的。但是呢呃如果我想要跟我认识的法国的同学交流的话，那可能还是会有一些小小的焦虑的。毕竟他是母语使用者。

提问：哦，ok，那就是你刚刚说就是你在呃因为你在学这个法语的时候就是有呃一点点焦虑，并不是很多。嗯，那你觉得就是这个一点点焦虑其实是不是跟这个难度有关系？比如说你学习更高level的法语，你可能到时候会会不会说觉得更加焦虑呢？

回答：呃，其实并不会的。因为我觉得作为语言的学习的话，其实它是有一个循序渐进的过程。那我们在打好基础的呃的前提下，一步一步的往上学，其实是不会说产生很大的焦虑情绪的。

提问：那你就是呃比如说遇到这么一点点焦虑的时候，那你是如何去处理这种一点点焦虑的呢？就怎么样去平复你的心情？有没有什么方法呢？

回答：如果如果当时是我跟母语使用者，比如说跟我法国的同学啊。呃在交流的过程中产生这样子的情绪的话，那他可能会比较用英语。我们就用第二语言进行交流。对。呃，那如果是我个人在学习的过程中，说实话，其实我在日常生活中还是比较喜欢看一些法国的电影，呃，我比较喜欢听法国歌，然后这样其实可以帮助我很多。

提问：嗯，挺好的。那你相信你自己是可以成为一个很擅长学习法语的人吗？

回答：呃，其实是可以的，我觉得法语其实在入门不算是很难的一门语言啊。嗯，呃，但是呢学任何一门语言其实都是要需要花很多的时间、精力和努力，只要花费时间去学，我觉得是可以学好的。

提问：对。那如果比如说就呃你刚毕业刚学完法语那会儿，呃，用1~10分来评估你掌握这个法语的自信的程度。你能给自己打多少分呢？就一是最低，十是最高。

回答：嗯，大概是4~5分吧，毕竟我只是学了个入门，但如果只是在我学的那个基础上稍微加一点点的话，其实可以达到五分。嗯，但是如果你说让我讲那些很很难很复杂的，或者去听一些很难很复杂的可能，可能这个分数就要低一点点。嗯，对，要根据实际的难度去判定这一题。

提问：嗯，确实，那你刚刚就是在思考评估你自己的这个呃学习法语的能力的时候，你是通过什么样的因素去考虑呢？

回答：呃，觉得可能还是自己的一个对于这门语言的一个掌握，我觉得可能我更看重的是沟通和交流吧，对，沟通和交流的流畅。

提问：嗯，嗯，那你现在你觉得你的就是法语沟通和交流的能力能到什么level？比如说日常的交流啊，还是说更高一点学术上啊之类的

回答：嗯，可能还是还是局限在日常交流。对。嗯。嗯。ok。

那比如说如果将来也有学弟学妹，就是你的呃学生啊之类的，也是会出现这种用英法双语语学习法语的情况。你会向他们提供一些什么样的建议呢？

回答：呃，非常具体的建议可能我给不了，因为每一个人学习语言都有自己的一个学习的方法和习惯。嗯。嗯，但是我觉得的话，其实用第二和第三语言结合去学习第三语言比我们用母语去学习第三，第二种语言我觉得其实是会更好一点。因为如果我们用我们自己的母语去学习外语的话，可能我们会对自己的母语呃会有一定会比就会有比较强的一个依赖性。可能你在学习的过程中会不断的依靠翻译这一种比较传统的方式去进行学习。但如果我们使用第二语言去学习第三语言的话，其实这里是有一个的，我觉得这个对于我们来学习来说其实是有帮助的。对。呃，具体的话什么建议可能暂时是没有啊。

提问：哦，那你这个我觉得讲的特别好，我这后插一个小问题哈。就比如说我们来排一个序，因为你刚刚启发到我就是嗯如果说我们呃有三种，第一种就是用呃法语去学法语。第二种是用英法双语去学法语。第三种是用中文去学法语，你觉得你更prefer哪一种方式？就做一个排序这样子。

回答：呃，如果可以的话，我我想选择第二种英语，用英法双语去学法语。我觉得我当时那个法国老师用国际音标去教我们是我们的法语发音，这种方法其实是非常好用的。无论我们当当时班里的同学有日本的，有西班牙的，还是像我这种来自中国的学生。我们在学习的时候使用同一种国际音标去学习。其实这时这时呢就其实对于我们来说作为初学这是非常有帮助的。嗯，对，所以我我觉得用英法双语去学法语是最好的。

提问：ok，ok，好的，好的，嗯，那这就是我们的采访了，也非常谢谢你的参与，那我们的采访到此结束。
