# Supplementary material for: Anxiety and self-efficacy in Chinese international students’ L3 French learning with L2 English and L3 French
Source: Front Psychol. 2022 Dec 16;13:998536. doi: 10.3389/fpsyg.2022.998536 (PMC9800968; doi:10.3389/fpsyg.2022.998536)
Supplement: Supplementary file 7 [file Data_Sheet_7.DOCX]

题目:大学生英语课堂焦虑与效能感调查问卷

基本信息

您的性别是：

1. 男 B.女

.您的年龄是______

Anxiety

1. 我不担心法语课上会犯错

1非常同意？2同意？3既不同意也不反对？4反对？5非常反对

2.. 法语课上老师叫我时会发抖

1非常同意？2同意？3既不同意也不反对？4反对？5非常反对

3. 法语课上没听懂老师用法语说什么会感到害怕

1非常同意？2同意？3既不同意也不反对？4反对？5非常反对

4.即使上更多的法语课，我也不觉得受困扰？

1非常同意？2同意？3既不同意也不反对？4反对？5非常反对

5.上法语课时在想一些和课堂内容无关的事

1非常同意？2同意？3既不同意也不反对？4反对？5非常反对

6. 法语课上做没有准备的发言时感到恐慌？

1非常同意？2同意？3既不同意也不反对？4反对？5非常反对

7.我不懂为何有些人在法语课上会如此心烦不安？

1非常同意？2同意？3既不同意也不反对？4反对？5非常反对

8.在法语课上主动发言会使我感到槛尬？

1非常同意？2同意？3既不同意也不反对？4反对？5非常反对

9.和外国人说法语不感到紧张？

1非常同意？2同意？3既不同意也不反对？4反对？5非常反对

10.经常感觉不想去上法语课

1非常同意？2同意？3既不同意也不反对？4反对？5非常反对

11.在法语课上发言很自信

1非常同意？2同意？3既不同意也不反对？4不同意？5非常反对

12. 法语老师要纠正我错误时很害怕

1非常同意？2同意？3既不同意也不反对？4不同意？5非常反对

13.快被叫到回答问题时我会感到心跳得很厉害

1非常同意？2同意？3既不同意也不反对？4不同意？5非常反对

14.在其他同学面前说法语会很拘谨

1非常同意？2同意？3既不同意也不反对？4不同意？5非常反对

15.我上法语课比上其它课更紧张和不安

1非常同意？2同意？3既不同意也不反对？4不同意？5非常反对

16.在法语课上发言讲英语时会感到紧张和困惑

1非常同意？2同意？3既不同意也不反对？4不同意？5非常反对

17.学法语要学那么多规则使人头疼

1非常同意？2同意？3既不同意也不反对？4不同意？5非常反对

18.说法语时担心别的同学取笑

1非常同意？2同意？3既不同意也不反对？4不同意？5非常反对

19.和外国人在一起感到轻松自在

1非常同意？2同意？3既不同意也不反对？4不同意？5非常反对

20.老师问事先没有准备的问题时感到紧张

1非常同意？2同意？3既不同意也不反对？4不同意？5非常反对

Self-efficacy:

21 .我对法语课程抱着一份，“60分万岁”的态度

1非常同意？2同意？3既不同意也不反对？4不同意？5非常反对

22 .我认为自己具备学好法语的各项能力

1非常同意？2同意？3既不同意也不反对？4不同意？5非常反对

23.我相信自己能成为学法语的高手

1非常同意？2同意？3既不同意也不反对？4不同意？5非常反对

24.我对提高法语成绩很有把握

1非常同意？2同意？3既不同意也不反对？4不同意？5非常反对

25.在法文交谈和阅读中遇到生词时，我能够通过猜测达到交流和理解的目的

1非常同意？2同意？3既不同意也不反对？4不同意？5非常反对

26学过的法语，我总觉得掌握的东西并不多

1非常同意？2同意？3既不同意也不反对？4不同意？5非常反对

27.老师在课堂上分析难度较大的文章时，我都能理解透彻

1非常同意？2同意？3既不同意也不反对？4不同意？5非常反对
