# Supplementary material for: Anxiety and self-efficacy in Chinese international students’ L3 French learning with L2 English and L3 French
Source: Front Psychol. 2022 Dec 16;13:998536. doi: 10.3389/fpsyg.2022.998536 (PMC9800968; doi:10.3389/fpsyg.2022.998536)
Supplement: Supplementary file 8 [file Data_Sheet_8.DOCX]

A survey about the relationship between anxiety and self-efficacy

Basic information

Your gender is:

A Male B Female

Your age is______

Anxiety scale (5-point Likert scale) :

(1)

(2) I tremble when I know that I’m going to be called on in French class.

(3) It frightens me when I do not understand what the teacher is saying in French.

(4) It wouldn't bother me at all to take more French classes

(5) During French class, I find myself thinking about things that have nothing to do with the course.

(6) I start to panic when I have to speak without preparation in French class.

(7) I don't understand why some people get so upset over French class.

(8) It embarrasses me to volunteer answers in my French class.

(9) I would not be nervous speaking French with native speakers.

(10) I often feel like not going to my French class.

(11) I feel confident when I speak in f French class.

(12) I am afraid that my French teacher is ready to correct every mistake I make.

(13) I can feel my heart pounding when I'm going to be called on in French class.

(14) I feel very self-conscious about speaking French in front of other students.

(15) I feel more tense and nervous in my French class than in my other classes.

(16) I get nervous and confused when I am speaking in my French class.

(17) I feel overwhelmed by the number of rules you have to learn to speak French.

(18) I am afraid that the other students will laugh at me when I speak French.

(19) I would probably feel comfortable around native speakers of French.

(20) I get nervous when the French teacher asks questions that I haven't prepared in advance.

Self-efficacy scale (5-point Likert scale):

(21) I will be satisfied if I only pass the French exam with a score of 60.

(22) I believe I have each ability that learning French requires.

(23) I believe I will be good at learning French.

(24). I am confident in improving my French score.

(25) When I use French to communicate and read, I can achieve the aims of communication and understanding.

(26) I feel that I do not master a lot of knowledge about French

(27) When the teacher analyzes difficult articles in class, I can fully understand.
